# Variation in Arthroscopic Treatment of Discoid Lateral Meniscus and Postoperative Restrictions in Children: Results of a Multicenter Meniscus Study Group Survey

**DOI:** 10.1177/23259671251333107

**Published:** 2025-05-07

**Authors:** Pierre-Henri Heitz, Thierry Pauyo, Jennifer J. Beck, Emily L. Niu, R. Jay Lee, J. Lee Pace, Gregory A. Schmale, Sasha Carsen, Benton E. Heyworth, Meniscus Research Interest Group (RIG);PRiSM, Matthew Milewski, John A. Schlechter, Zachary S. Stinson, Mark Tompkins, Matthew J. Brown, Craig J. Finlayson, Philip L. Wilson, Jennifer Brey, Marie-Lyne Nault

**Affiliations:** Université de Montréal, Department of Surgery, Montréal, Quebec, Canada; CHU Sainte-Justine, Montréal, Quebec, Canada; Canada Shriners Hospital, Montréal, Quebec, Canada; Canada Shriners Hospital, Montréal, Quebec, Canada; Children’s National Hospital, Washington, DC, USA; Johns Hopkins Hospital, Baltimore, Maryland, USA; Elite Sports Medicine, Connecticut Children's Medical Center, Farmington, Connecticut, USA; Seattle Children's and University of Washington School of Medicine, Seattle, Washington, USA; Children’s Hospital of Eastern Ontario, Ottawa, Ontario, Canada; Boston Children's Hospital, Boston, Massachusetts, USA; Harvard Medical School, Boston, Massachusetts, USA; Department of Orthopaedic Surgery, Boston Children's Hospital, Boston, Massachusetts, USA; Pediatric Orthopedic Specialists Orange County, Children's Hospital of Orange County, Orange, California, USA; Nemours Children's Health, Orlando, Florida, USA; University of Minnesota, Minneapolis, Minnesota, USA; Orthopedics and Sports Medicine at Connecticut Children's, Wesport Connecticut, USA; Ann and Robert H. Lurie Children's Hospital, Chicago, Illinois, USA; Scottish Rite for Children, Dallas, Texas, USA; Department of Orthopaedic Surgery, University of Louisville, Louisville, Kentucky, USA; Université de Montréal, Department of Surgery, Montréal, Quebec, Canada; CHU Sainte-Justine, Montréal, Quebec, Canada; Canada Shriners Hospital, Montréal, Quebec, Canada; CIUSSS-NIM, Hôpital du Sacré-Cœur de Montréal, Montréal, Quebec, Canada; Investigation performed at CHU Sainte-Justine, Montréal, Quebec, Canada

**Keywords:** discoid lateral meniscus, intersurgeon variability, operative planning, survey, surgical treatment, postoperative planning

## Abstract

**Background:**

Because of congenital abnormal collagenous structures and peripheral attachments, discoid lateral meniscus (DLM) is often associated with tears and instability and a risk of premature compartmental articular degeneration. Typically, surgery is indicated for symptomatic patients. Several surgical techniques have been described in the literature, but no studies have examined surgeon variation.

**Purpose:**

To determine the intersurgeon agreement for DLM surgical planning and postoperative restriction after arthroscopic assessment.

**Study Design:**

Cross-sectional study.

**Methods:**

Sixteen orthopaedic surgeons from an international pediatric meniscus study group were shown 4 different videos of a DLM arthroscopic procedure. Each surgeon completed a survey for each arthroscopic video. The survey included questions about operative planning, such as peripheral rim preservation, repair and stabilization technique, and postoperative restrictions. Descriptive statistics were used to characterize the variables collected.

**Results:**

One of the 4 arthroscopic videos showed a complete discoid meniscus with no instability or tearing. For this specific meniscus, consensus was generally good, with 87.5% to 93.8% agreement for all the categories, except peripheral rim preservation, with 31.3% agreement. However, for the other 3 videos with tears and/or instability, consensus was generally low. Depending on the categories, the highest percentage of intersurgeon agreement varied between 25.0% and 62.5%, while being >50% only 2 of 27 times.

**Conclusion:**

Agreement regarding the surgical technique and postoperative rehabilitation among surgeons with experience in the treatment of DLM is poor in the event of tears and instability, which represent the greatest technical challenges with the most significant functional effect. Given the relative rarity of the condition and the implications on a child's long-term joint health, prospective, comparative multicenter studies regarding treatment algorithms and outcomes are warranted.

Discoid lateral meniscus (DLM) is a congenital variant present in approximately 0.5% to 17.0% of the population.^8,24^ DLM is typically characterized by a wider meniscus covering a larger portion of the tibial plateau. Its unusual thickness, which may alter biomechanics and force distribution, combined with an altered collagenous structure and vascularity, is correlated with tears and premature chondral degenerative diseases.^4,7^ Diagnosis of DLM is usually made by combining physical examination, imaging, and, if indicated, arthroscopy. Clinical presentation is highly variable. Some DLMs are asymptomatic, whereas others will present with pain, limited range of motion, or a reproducible clunk.^8,23^ Diagnosis is then confirmed with magnetic resonance imaging and/or arthroscopy.^29^

Symptomatic DLMs presenting with instability and/or tears are often eligible for surgery. Arthroscopic surgeons agree that saucerization should be performed while preserving the natural meniscal rim as much as possible, to lower the risk of developing degenerative disease.^2,12^ However, a wide variety of procedures and surgical techniques exist, and there is no definitive agreement in the literature on which is most effective. In a recent systematic review, 5 studies were analyzed regarding the benefit of meniscal repair in patients with DLM. Smuin et al^25^ did not advocate meniscal repair after saucerization, while other studies recommended meniscal repair, as needed, after partial meniscectomy for optimal outcomes.^2,21^ Other topics, such as a leaflet-sparing technique, peripheral rim preservation threshold, or the type of suture technique used, vary widely between studies and are subject to debate.^6,14,26,27^

Many published case series have demonstrated the differences in DLM surgical techniques and protocols. However, they only showed variability between different groups and did not examine the variability within a cohort of ultra-specialized sports medicine surgeons with a specific interest in the pediatric meniscus, its associated conditions, and surgery. The purpose of the present study was primarily to determine the intersurgeon agreement in the choice of DLM surgical technique based on an arthroscopic assessment and secondarily to determine intersurgeon agreement in postoperative restriction after DLM surgery. We hypothesized that agreement would be poor between surgeons regarding operative technique as well as postoperative recommendations.

## Methods

A unique research survey was created and approved by our institution's ethics committee before being completed by surgeons between February and July 2021. Orthopaedic surgeons trained and specialized in pediatrics and/or sports medicine and part of the Pediatric Research in Sport Medicine (PRiSM) Meniscus Research Interest Group (RIG) were asked to participate in this study. Each orthopaedic surgeon was provided with 4 de-identified videos of a DLM diagnostic arthroscopic procedure. The 4 videos were chosen from a database created by the PRiSM RIG focusing on the meniscus. The only pathology found in all the videos was discoid meniscus. The choice of arthroscopic videos for the survey was based on the 2 following criteria: (1) video quality had to be excellent to limit any variability in interpretation, and (2) the videos were selected to represent a broad spectrum of discoid pathologies based on the PRiSM classification.^13^ The videos were classified by the PRiSM RIG. Reviewers were blinded to patient characteristics such as age, sex, body mass index, duration of symptoms, or date of onset. These characteristics were withheld to remove any confounding factors that could potentially influence surgeons’ decision-making.^9,10,32^

The selected arthroscopic assessment videos were taken by experienced orthopaedic surgeons, and all assessments followed the same written protocol. The procedure was standardized with the use of anteromedial and anterolateral portals. The arthroscope was inserted through the anterolateral portal for viewing and initial probing, and inspection was performed through the anteromedial portal to evaluate overall meniscal size as well as stability of the posterior horn and body. A thorough exploration of the lateral intra-articular compartment using the probe was done for the purpose of determining DLM thickness and width, as well as the presence of meniscal hypermobility or instability. To evaluate the anterior horn, the arthroscope was placed through the anteromedial portal and the probe through the anterolateral portal, where anterior horn stability could be properly assessed. While not part of the final video, saucerization was then performed and the evidence or absence of tear in the residual meniscus was assessed. A postsaucerization assessment of the meniscus was performed in a similar manner at the end of the video.

**Figure 1.**
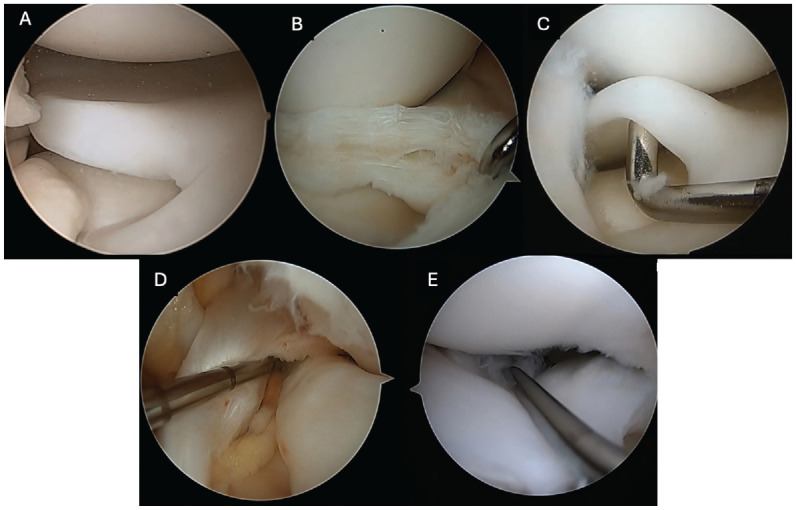
Images from arthroscopic videos of different discoid lateral menisci. (A) From the first video, classified as W2;H0;S0;T0. (C) From the second video, classified as W2;H0;SA;T0. (B and D) From the third video, classified as W2;H1;S0;THAP, with B showing the horizontal tear after saucerization was completed and D showing the height and width. (E) From the fourth video, classified as W2;H1;SP;THAP, showing posterior horn instability.

Based on the PRiSM classification^13^ (Table 1), the first video demonstrated >90% coverage of the tibial plateau by the meniscus without increased height, instability, or tear (W2;H0;S0;T0). The second video showed >90% coverage of the tibial plateau by the meniscus with anterior meniscocapsular instability but without increased height and tear (W2;H0;SA;T0). The third video displayed >90% coverage of the tibial plateau by the meniscus with increased height and anteroposterior tear (central horizontal cleavage) but without instability (W2;H1;S0;THAP). The last video presented a >90% coverage of the tibial plateau by the meniscus with increased height, posterior meniscocapsular instability, and anteroposterior tear (horizontal cleavage) (W2;H1;SP;THAP) (Figure 1).

**Table 1:** PRiSM Classification^13^

Class	Description
Width	
W0	Normal
W1	Incomplete
W2	Near complete/complete
Height	
H0	Normal
H1	Abnormal
Stability	
S0	Normal
SA	Abnormal anterior
SP	Abnormal posterior
SAP	Abnormal anteroposterior
Nonvertical tears	
T0	Normal or central tear
THA	Horizontal anterior tear
THP	Horizontal posterior tear
THAP	Horizontal anteroposterior tear
TDA	Degenerative complex anterior tear
TDP	Degenerative complex posterior tear
TDAP	Degenerative complex anteroposterior tear

Reviewers completed a survey for each arthroscopic video (16 surgeons × 4 videos = 64 surveys total). Orthopaedic surgeons’ experience and training were recorded at the beginning of the study. Questions on the influence of factors such as weight, body mass index, duration of symptoms, age of onset, and severity of cartilage lesion on preoperative planning or postoperative restriction protocols were completed. After watching the arthroscopic videos, multiple-choice questions regarding the surgical method (peripheral rim preservation, repair techniques, and tissue repair-stimulating techniques) and postoperative protocols (weightbearing and range of motion) were completed. It was possible to select >1 answer for the multiple-choice questions and select the answer labeled as “other,” in which the surgeon could write what he or she would have done specifically. If >1 answer was chosen, it was recorded as combined (Appendix 1).

### Statistical Analysis

Descriptive statistics (mean and standard deviation) are used to characterize the surgeons and compare variables collected to determine the level of consensus between orthopaedic surgeons. Percentages are used to present how the answers were distributed. If a similar answer was selected X% of the time, it is possible to say that agreement between the group of orthopaedic surgeons was reached X% of the time.

## Results

Sixteen orthopaedic surgeons, of the 19 members of the PRiSM Meniscus RIG targeted, answered the survey for this study. Among them, 78.6% had completed fellowships in sports medicine and pediatric orthopaedics, while 14.3% had completed a fellowship only in pediatric orthopaedics, and 7.1% of them had between 5 and 10 years of experience, while 35.7% had >10 years of experience. The most significant factors to determine postoperative activity restrictions were severity of the associated cartilage lesion and patient age, which were selected 50.0% and 20.0% of the time, respectively, by the participants. It is important to note that this is only an association and does not provide information on whether there is a correlation or its direction.

Considering all 9 categories of the survey, the mean maximum agreement between orthopaedic surgeons for the first video (W2;H0;S0;T0) was 83.3% (SD, 19.8%). The mean agreement was lower for the subsequent videos, with 41.0% (SD, 8.9%) for the second video (W2;H0:SA;T0), 39.6% (SD, 11.7%) for the third video (W2;H1;S0;THAP), and 41.7% (SD, 10.8%) for the fourth video (W2;H1;SP;THAP).

**Figure 2.**
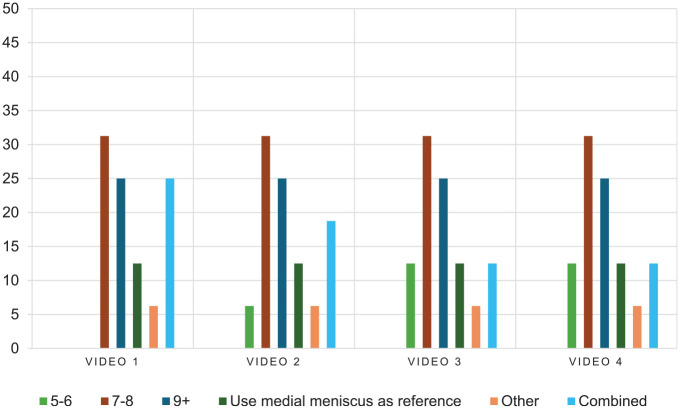
Bar graph presenting the distribution of surgeon answers for peripheral rim preservation. The Y axis represents the distribution of answers in percentage. The scales for the bar graph are different and range from 0% to 50%. Values for rim preservation are expressed in millimeters. Video 1 corresponds to W2;H0;S0;T0, video 2 corresponds to W2;H0;SA;T0, video 3 corresponds to W2;H1;S0;THAP, and video 4 corresponds to W2;H1;SP;THAP.

Based on descriptive statistics, agreement between surgeons was generally good to excellent in the first video (W2;H0;S0;T0), showing a complete discoid meniscus with normal height and no instability or tears. For all categories (operative management and rehabilitation), 87.5% to 93.8% of the orthopaedic surgeons selected similar answers and, therefore, seemed to agree with each other. There was a lack of consensus only for the peripheral rim preservation category, which was the only abnormality to address in this specific video. The highest level of agreement was 31.3% (peripheral rim preservation of 7-8 mm), which was comparable to the other videos (Figure 2).

**Figure 3.**
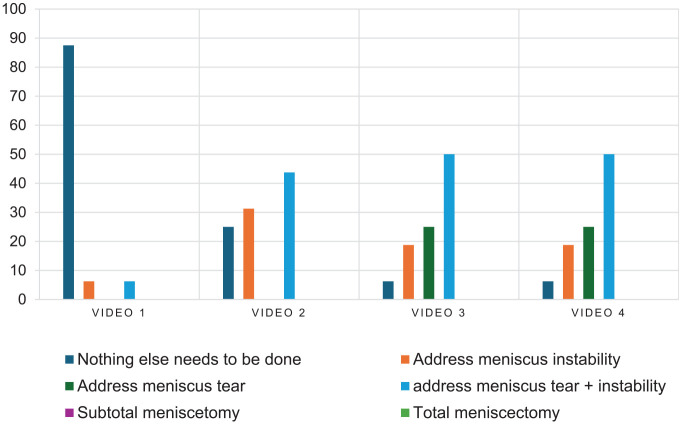
Bar graph presenting the distribution of surgeon answers for operative planning. The Y axis represents the distribution of answers in percentage. Video 1 corresponds to W2;H0;S0;T0, video 2 corresponds to W2;H0;SA;T0, video 3 corresponds to W2;H1;S0;THAP, and video 4 corresponds to W2;H1;SP;THAP.

**Figure 4.**
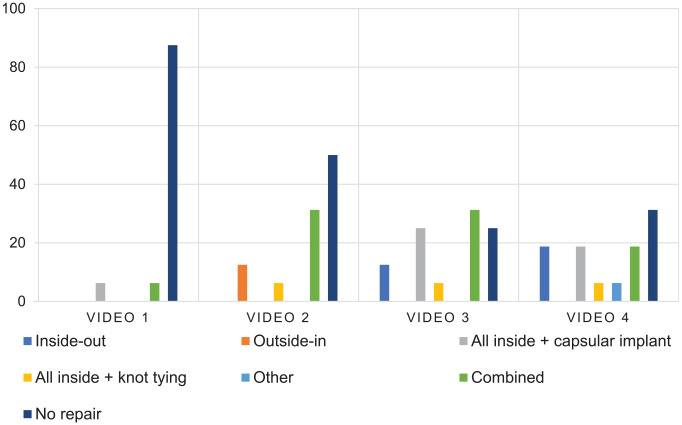
Bar graph presenting the distribution of surgeon answers for meniscal tear repair technique. The Y axis represents the distribution of answers in percentage. Video 1 corresponds to W2;H0;S0;T0, video 2 corresponds to W2;H0;SA;T0, video 3 corresponds to W2;H1;S0;THAP, and video 4 corresponds to W2;H1;SP;THAP.

In the second video (W2;H0;SA;T0), agreement varied between 25.0% and 50.0%, depending on the category (Figures 3 and 4). Fifty percent agreement was reached for the following categories: meniscal repair technique, the number of sutures for repair, and postoperative ROM restriction. The lowest level of agreement was for the number of sutures for instability.

In the third video (W2;H1;S0;THAP), agreement varied between 25% and 62.5%, depending on the category: 62.5% for the postoperative ROM restriction category and 25% for the meniscal repair technique category.

In the fourth video (W2;H1;SP;THAP), agreement varied between 31.3% and 62.5%, depending on the category: 62.5% for the postoperative ROM restriction category and the lowest level of agreement for peripheral rim preservation, meniscal repair technique, and the number of sutures for repair.

Peripheral rim preservation was the only category for which surgeon agreement never exceeded 33.1% across all 4 arthroscopic videos (Figure 2). When excluding the first video, where there was no instability or tear, only postoperative range of motion restriction had a level of agreement >50%. Agreement among surgeons was >50% only twice when the first video (W2;H0;So;T0) was excluded, and it was ≤50% for the 25 other questions. The highest intersurgeon agreement for the different categories of operative planning and postoperative restrictions is presented in Table 2, and a more detailed presentation of intersurgeon agreement is presented in Appendix 2.

**Table 2:** Presentation of the Category With the Highest Intersurgeon Agreement for Each Question Regarding Operative Planning and Postoperative Restrictions^*a*^

Category	Video 1	Video 2	Video 3	Video 4
After saucerization, what needs to be addressed	Nothing	Tear and instability	Tear and instability	Tear and instability
	87.5%	43.8%	50.0%	50.0%
Peripheral rim preservation	6-8 mm	6-8 mm	6-8 mm	6-8 mm
	31.3%	31.3%	31.3%	31.3%
Meniscal repair technique	No repair	No repair	Combined technique	No repair
	87.5%	50.0%	25.0%	31.3%
No. of sutures for repair technique	No suture	No suture	3-4	No suture or 5-6
	87.5%	50.0%	31.3%	31.3%
Meniscal instability technique	No instability	Anterior outside-in	Posterior all-inside	Combined technique
	87.5%	37.5%	43.8%	43.8%
No. of sutures for instability technique	No instability	1-2 or 3-4 or no suture	No instability	No instability
	87.5%	25.0%	31.3%	37.5%
Repair-stimulating technique	No stimulating technique	Combined technique	Combined technique	Combined technique
	93.8%	43.8%	43.8%	50.0%
Weightbearing	FWB	PWB or FWB	FWB	PWB or FWB
	93.8%	37.5%	37.5%	37.5%
ROM	Full ROM	Full ROM	Restricted ROM	Restricted ROM
	93.8%	50.0%	62.5%	62.5%
Highest mean level of agreement	83.3%	41.0%	39.6%	41.7%
SD	19.8%	8.9%	11.7%	10.8%

a When “or” is used in the table, this means that ≥2 answers presented the highest agreement among surgeons. Video 1 corresponds to W2;H0;S0;T0, video 2 corresponds to W2;H0;SA;T0, video 3 corresponds to W2;H1;S0;THAP,and video 4 corresponds to W2;H1;SP;THAP. FWB, full weightbearing; PWB, partial weightbearing; ROM, range of motion.

## Discussion

The present study highlights the variations in responses among surgeons to the operative and postoperative strategies regarding DLM. Agreement among surgeons was rarely >50% for specific questions proposed in the survey, particularly in the presence of more complex DLM pathologies (eg, presence of instability or tears). Surgeons tended to agree with each other when there was no tear or instability. Therefore, as the pathology became more complex, variability in surgeon strategy appeared to increase as well.

Recent studies suggest an approach in which peripheral rim preservation is key to preventing long-term complications such as early degenerative changes.^1,12,17,25,33^ Despite having agreed that peripheral preservation is important, how much to preserve is debatable. None of the studies published have determined the specific rim width that should be kept to achieve the perfect balance between symptom relief and prevention of recurrence, as well as knee stability and prevention of degenerative articular changes. The most commonly agreed on rim preservation size found in the literature is 6 to 8 mm,^1,2,7,11^ although some studies recommend as little as 4 to 5 mm,^7^ while others recommend at least 10 mm.^5,18^ Nishino et al^18^ favor 10 mm after their observation of a significant decrease in meniscal width 3 to 24 months postoperatively. Therefore, keeping 7 to 8 mm of the rim may not suffice to prevent knee osteoarthritis. More recently, Gamble et al^5^ suggested that peripheral rim preservation should be up to 16 mm, which was determined to be the mean width of an adult meniscus. However, theirs was a biomechanical study, which did not present clinical results or take into consideration other factors such as tears, magnitude of meniscal degeneration, and patient symptoms. When it came to rim preservation, the answers to the survey were quite heterogeneous, but most surgeons chose a size ≥7 to 8 mm (80% of responders). Therefore, despite intersurgeon variability in terms of peripheral preservation, overall, the scientific recommendations of preserving at least 6 mm were followed.

There are different guidelines when it comes to DLM treatment. Most recommend repairing tears when a tear is present and stabilizing the meniscus when instability is present.^7,19,23^ In the current study, surgeons did not agree on whether to repair meniscal tears and/or to address meniscal instability. For this specific question, intersurgeon agreement never exceeded 50%, with some surgeons wanting to only repair meniscal tears, some wanting to only address meniscal instability, others wanting to do both, and still others not wanting to do either. Variability in DLM classification and diagnosis may explain this lack of agreement. However, a recent study described a new classification method with moderate to substantial interrater agreement for DLM classification, all categories considered.^13^ Additionally, despite surgical recommendations found in guidelines, there is still heterogeneity and variability within techniques and surgeons’ skill sets. Smuin et al^25^ reported that meniscal repair had little to no effect on postoperative outcomes, while Lins et al^16^ found that frequency of meniscal revision did not differ whether repair was done or not. Lastly, tactile feedback is important when assessing instability and tears, and limiting surgeons to watching videos, rather than physically probing and assessing the meniscus, may have impacted their decision-making.

These results do raise clinical concerns as variability in treatment may result in variability in patient outcomes. Many categories for which surgeons were not in agreement, such as the type of suture or the number of sutures, are not clinically significant.^15,21,33^ However, other categories (eg, “Should the tear or instability be addressed or not?”) could have more effect on patient outcomes. These concerns extend to postoperative care. High variability from strict immobilization and nonweightbearing to full range of motion and full weightbearing can affect outcomes, patient satisfaction, and compliance in the postoperative period. With restrictions, there is a risk for muscle atrophy and ankylosis, while not enough restrictions may alter tissue healing.^28,29,30^

At the time of writing, there were no other studies published on the variability in surgical treatment for meniscal tears. However, we found other publications describing variations in treatment for other pathologies. Aubin et al^3^ described an important intersurgeon variability in surgical planning for scoliosis regarding type of screws, numbers of rods and screws, vertebral level, and many more categories. Parsons et al^20^ reported a high variability for implant orientation (ICC = 0.37), size (ICC = 0.36), and type (ICC = 0.36) in shoulder arthroplasty, while Petrera et al^22^ found that intersurgeon reliability for suture placement for Bankart lesion repair was only fair (ICC, 0.40). This is not unique to orthopaedic surgery. Indeed, a recent study reported high intersurgeon variability regarding lymph node excision during laparoscopic cholecystectomy and a lack of standardization.^31^ Research focusing on surgical decision-making and its effect on postoperative outcomes must continue in all surgical specialties.

### Limitations

There are some limitations to our study. First, the small number of videos (n = 4) limited our ability to analyze interrater reliability with only descriptive statistics presented. The number of cases was limited to improve survey completion rates. Additionally, the significant heterogeneity of responses limited the utility of further statistical analysis. Second, surgeons were shown a DLM arthroscopic video performed by a different surgeon, with the purpose of limiting potential confounding factors and bias. However, it is important to note that a simple video representation does not fully capture the complexity of real-life surgical experiences. Surgeons must consider various factors such as patient-specific characteristics, symptoms, physical examinations, and tactile feedback during surgery. The absence of tactile sensation and the inability to physically manipulate the meniscus in the video format may limit the accuracy of surgical decision-making. It is possible that survey responses could have differed if conducted in a real operating room setting, where surgeons have the opportunity to rely on tactile sensation, probing, and other methods to judge the situation more accurately. Thus, it is likely that agreement would be higher in a more realistic clinical and surgical context. Another limitation is the subjectivity when interpreting the various survey questions and responses, which is inherent to this study design. It is possible that this subjectivity may artificially amplify variability among responses, even though surgeons might ultimately choose a similar course of action when faced with a similar case. To minimize any interpretation bias, we provided a wide range of response options in the survey and allowed surgeons the opportunity to provide additional comments or alternatives. Additionally, the responses regarding intact meniscus scenarios showed little variation, suggesting potentially reduced subjectivity. Nonetheless, to our knowledge, this is the first study to have examined surgeon agreement in DLM surgical planning; no other study has determined the specific effect of patients’ symptoms as well as the difference between the surgeon doing the arthroscopy and a surgeon watching a video of an arthroscopy on the level of agreement in DLM surgical planning.

## Conclusion

Our study highlights the variability among surgeons regarding operative and postoperative planning for the treatment of DLM. This variability emphasizes the complexity of addressing this condition. Further studies, including large retrospective and prospective studies examining the outcomes of various treatment algorithms, as well as the use of a standardized DLM classification system, will shed more light on the optimal treatment for DLM.

## Authors

Pierre-Henri Heitz, MD (Université de Montréal, Department of Surgery, Montréal, Quebec, Canada; CHU Sainte-Justine, Montréal, Quebec, Canada); Thierry Pauyo, MD (Canada Shriners Hospital, Montréal, Quebec, Canada); Jennifer J. Beck (Canada Shriners Hospital, Montréal, Quebec, Canada); Emily L. Niu, MD (Children’s National Hospital, Washington, DC, USA); R. Jay Lee, MD (Johns Hopkins Hospital, Baltimore, Maryland, USA); J. Lee Pace (Elite Sports Medicine, Connecticut Children's Medical Center, Farmington, Connecticut, USA); Gregory A. Schmale, MD (Seattle Children's and University of Washington School of Medicine, Seattle, Washington, USA); Sasha Carsen, MD, MBA (Children’s Hospital of Eastern Ontario, Ottawa, Ontario, Canada); Benton E. Heyworth, MD (Boston Children's Hospital, Boston, Massachusetts, USA; Harvard Medical School, Boston, Massachusetts, USA); PRiSM Meniscus Research Interest Group (RIG); Matthew Milewski, MD (Department of Orthopaedic Surgery, Boston Children's Hospital, Boston, Massachusetts, USA); John A. Schlechter, MD (Pediatric Orthopedic Specialists Orange County, Children's Hospital of Orange County, Orange, California, USA); Zachary S. Stinson, MD (Nemours Children's Health, Orlando, Florida, USA); Mark Tompkins, MD (University of Minnesota, Minneapolis, Minnesota, USA); Matthew J. Brown, MD (Orthopedics and Sports Medicine at Connecticut Children's, Wesport Connecticut, USA); Craig J. Finlayson, MD (Ann and Robert H. Lurie Children's Hospital, Chicago, Illinois, USA); Philip L. Wilson, MD (Scottish Rite for Children, Dallas, Texas, USA); Jennifer Brey, MD (Department of Orthopaedic Surgery, University of Louisville, Louisville, Kentucky, USA); and Marie-Lyne Nault, MD, PhD (Université de Montréal, Department of Surgery, Montréal, Quebec, Canada; CHU Sainte-Justine, Montréal, Quebec, Canada; Canada Shriners Hospital, Montréal, Quebec, Canada; CIUSSS-NIM, Hôpital du Sacré-Cœur de Montréal, Montréal, Quebec, Canada).

## Supplemental Material

sj-docx-1-ojs-10.1177_23259671251333107 – Supplemental material for Variation in Arthroscopic Treatment of Discoid Lateral Meniscus and Postoperative Restrictions in Children: Results of a Multicenter Meniscus Study Group Survey Supplemental material, sj-docx-1-ojs-10.1177_23259671251333107 for Variation in Arthroscopic Treatment of Discoid Lateral Meniscus and Postoperative Restrictions in Children: Results of a Multicenter Meniscus Study Group Survey by Pierre-Henri Heitz, Thierry Pauyo, Jennifer J. Beck, Emily L. Niu, R. Jay Lee, J. Lee Pace, Gregory A. Schmale, Sasha Carsen, Benton E. Heyworth, Matthew Milewski, John A. Schlechter, Zachary S. Stinson, Mark Tompkins, Matthew J. Brown, Craig J. Finlayson, Philip L. Wilson, Jennifer Brey and Marie-Lyne Nault in Orthopaedic Journal of Sports Medicine

## References

[1] Ahn JH Kang DM Choi KJ. Risk factors for radiographic progression of osteoarthritis after partial meniscectomy of discoid lateral meniscus tear. Orthop Traumatol Surg Res. 2017;103(8):1183-1188. 28987527 10.1016/j.otsr.2017.09.013

[2] Ahn JH Kim KI Wang JH Jeon JW Cho YC Lee SH. Long-term results of arthroscopic reshaping for symptomatic discoid lateral meniscus in children. Arthroscopy. 2015;31(5):867-873. 25665957 10.1016/j.arthro.2014.12.012

[3] Aubin CE Labelle H Ciolofan OC. Variability of spinal instrumentation configurations in adolescent idiopathic scoliosis. Eur Spine J. 2007;16(1):57-64. 16477449 10.1007/s00586-006-0063-6 PMC2198894

[4] Choi YH Seo YJ Ha JM Jung KH Kim J Song SY. Collagenous ultrastructure of the discoid meniscus: a transmission electron microscopy study. Am J Sports Med. 2017;45(3):598-603. 27899354 10.1177/0363546516674181

[5] Gamble JG Abdalla AB Meadows M , et al. Lateral meniscus width at the popliteus recess and the relevance to saucerization of discoid lateral menisci. Orthop J Sports Med. 2020;8(4 suppl 3):2325967120S00228.

[6] Haemer JM Wang MJ Carter DR Giori NJ. Benefit of single-leaf resection for horizontal meniscus tear. Clin Orthop Relat Res. 2007;457:194-202. 17179782 10.1097/BLO.0b013e3180303b5c

[7] Kim JH Ahn JH Kim JH Wang JH. Discoid lateral meniscus: importance, diagnosis, and treatment. J Exp Orthop. 2020;7(1):81. 33044686 10.1186/s40634-020-00294-y PMC7550551

[8] Kocher MS Logan CA Kramer DE. Discoid lateral meniscus in children: diagnosis, management, and outcomes. J Am Acad Orthop Surg. 2017;25(11):736-743. 29059110 10.5435/JAAOS-D-15-00491

[9] Kose O Celiktas M Egerci OF Guler F Ozyurek S Sarpel Y. Prognostic factors affecting the outcome of arthroscopic saucerization in discoid lateral meniscus: a retrospective analysis of 48 cases. Musculoskelet Surg. 2015;99(2):165-170. 25986993 10.1007/s12306-015-0376-x

[10] Lau BC Vashon T Janghala A Pandya NK. The sensitivity and specificity of preoperative history, physical examination, and magnetic resonance imaging to predict articular cartilage injuries in symptomatic discoid lateral meniscus. J Pediatr Orthop. 2018;38(9):e501-e506. 10.1097/BPO.0000000000001221 30036288

[11] Lee CR Bin SI Kim JM Lee BS Kim NK. Arthroscopic partial meniscectomy in young patients with symptomatic discoid lateral meniscus: an average 10-year follow-up study. Arch Orthop Trauma Surg. 2018;138(3):369-376. 29188421 10.1007/s00402-017-2853-1

[12] Lee DH D’Lima DD Lee SH. Clinical and radiographic results of partial versus total meniscectomy in patients with symptomatic discoid lateral meniscus: a systematic review and meta-analysis. Orthop Traumatol Surg Res. 2019;105(4):669-675. 31027980 10.1016/j.otsr.2019.02.023

[13] Lee RJ Nepple JJ Schmale GA , et al. Reliability of a new arthroscopic discoid lateral meniscus classification system: a multicenter video analysis. Am J Sports Med. 2022;50(5):1245-1253. 35234542 10.1177/03635465221076857

[14] Lee SW Chun YM Choi CH , et al. Single-leaf partial meniscectomy in extensive horizontal tears of the discoid lateral meniscus: does decreased peripheral meniscal thickness affect outcomes? (Mean four-year follow-up). Knee. 2016;23(3):472-477. 26875744 10.1016/j.knee.2015.11.019

[15] Lee YS Teo SH Ahn JH Lee OS Lee SH Lee JH. Systematic review of the long-term surgical outcomes of discoid lateral meniscus. Arthroscopy. 2017;33(10):1884-1895. 28655477 10.1016/j.arthro.2017.04.006

[16] Lins LAB Feroe AG Yang B , et al. Long-term minimum 15-year follow-up after lateral discoid meniscus rim preservation surgery in children and adolescents. J Pediatr Orthop. 2021;41(9):e810-e815. 10.1097/BPO.0000000000001903 34411050

[17] Mochizuki T Tanifuji O Sato T Watanabe S Endo N. Predictive factors for developing osteochondritis dissecans after surgery for discoid lateral meniscus are younger age and shorter meniscal width. Knee Surg Sports Traumatol Arthrosc. 2021;29(1):100-108. 31642945 10.1007/s00167-019-05750-6

[18] Nishino K Hashimoto Y Tsumoto S Yamasaki S Nakamura H. Morphological changes in the residual meniscus after reshaping surgery for a discoid lateral meniscus. Am J Sports Med. 2021;49(12):3270-3278. 34415178 10.1177/03635465211033586

[19] Ohnishi Y Nakashima H Suzuki H Nakamura E Sakai A Uchida S. Arthroscopic treatment for symptomatic lateral discoid meniscus: the effects of different ages, groups and procedures on surgical outcomes. Knee. 2018;25(6):1083-1090. 30115588 10.1016/j.knee.2018.06.003

[20] Parsons M Greene A Polakovic S , et al. Intersurgeon and intrasurgeon variability in preoperative planning of anatomic total shoulder arthroplasty: a quantitative comparison of 49 cases planned by 9 surgeons. J Shoulder Elbow Surg. 2020;29(12):2610-2618. 33190760 10.1016/j.jse.2020.04.010

[21] Perkins CA Busch MT Christino MA Willimon SC. Saucerization and repair of discoid lateral menisci with peripheral rim instability: intermediate-term outcomes in children and adolescents. J Pediatr Orthop. 2021;41(1):23-27. 33044260 10.1097/BPO.0000000000001695

[22] Petrera M Ogilvie-Harris DJ Theodoropoulos JS , et al. Inter-surgeon variability in the identification of clock face landmarks when placing suture anchors in arthroscopic Bankart repair. Shoulder Elbow. 2018;11(6):419-423. 32269601 10.1177/1758573218797964 PMC7094062

[23] Saavedra M Sepúlveda M Jesús Tuca M Birrer E. Discoid meniscus: current concepts. EFORT Open Rev. 2020;5(7):371-379. 32818064 10.1302/2058-5241.5.190023 PMC7407869

[24] Smith RA Vandenberg CD Pace JL. Management of long-term complications in the setting of lateral meniscal deficiency after saucerization of a discoid lateral meniscus in an adolescent patient: a case report and review of the literature. JBJS Case Connect. 2018;8(4):e102. 10.2106/JBJS.CC.18.00054 30540611

[25] Smuin DM Swenson RD Dhawan A. Saucerization versus complete resection of a symptomatic discoid lateral meniscus at short- and long-term follow-up: a systematic review. Arthroscopy. 2017;33(9):1733-1742. 28865577 10.1016/j.arthro.2017.03.028

[26] Strauss EJ Jazrawi LM. The Management of Meniscal Pathology. Springer International Publishing; 2020.

[27] Tsujii A Matsuo T Kinugasa K Yonetani Y Hamada M. Arthroscopic minimum saucerization and inferior-leaf meniscectomy for a horizontal tear of a complete discoid lateral meniscus: report of two cases. Int J Surg Case Rep. 2018;53:372-376. 30481736 10.1016/j.ijscr.2018.11.027 PMC6260367

[28] van Wijk L van Duinhoven S Liem MSL Bouman DE Viddeleer AR Klaase JM. Risk factors for surgery-related muscle quantity and muscle quality loss and their impact on outcome. Eur J Med Res. 2021;26(1):36. 33892809 10.1186/s40001-021-00507-9 PMC8063361

[29] Watanabe M TS Ikeuchi HJ. Atlas of Arthroscopy. 2nd ed. Igaku-Shoin; 1969.

[30] Wiley TJ Lemme NJ Marcaccio S , et al. Return to play following meniscal repair. Clin Sports Med. 2020;39(1):185-196. 31767105 10.1016/j.csm.2019.08.002

[31] Wysocki AP Murphy S Baade I. Inter-surgeon variability in cystic artery lymph node excision during laparoscopic cholecystectomy. Cureus. 2018;10(6):e2759-e. 10.7759/cureus.2759 PMC6080738 30094116

[32] Yang SJ Li J Xue Y Zhang Z Chen G. Multivariate ordered logistic regression analysis of the postoperative effect of symptomatic discoid lateral meniscus. Arch Orthop Trauma Surg. 2021;141(11):1935-1944. 33616721 10.1007/s00402-021-03821-3 PMC8497286

[33] Yoo WJ Jang WY Park MS , et al. Arthroscopic treatment for symptomatic discoid meniscus in children: midterm outcomes and prognostic factors. Arthroscopy. 2015;31(12):2327-2334. 26321109 10.1016/j.arthro.2015.06.032

