# Supplementary material for: Variation in Arthroscopic Treatment of Discoid Lateral Meniscus and Postoperative Restrictions in Children: Results of a Multicenter Meniscus Study Group Survey
Source: Orthop J Sports Med. 2025 May 7;13(5):23259671251333107. doi: 10.1177/23259671251333107 (PMC12062721; doi:10.1177/23259671251333107)
Supplement: sj-docx-1-ojs-10.1177_23259671251333107 – Supplemental material for Variation in Arthroscopic Treatment of Discoid Lateral Meniscus and Postoperative Restrictions in Children: Results of a Multicenter Meniscus Study Group Survey [file sj-docx-1-ojs-10.1177_23259671251333107.docx]

Appendix 1: Survey presented to the orthopaedic surgeons (officially done using google form)

**Inter-surgeon variability in DLM pre-op management and post-op restrictions:**

*Question 1 – 5 are to be answered once and question 6 – 15 are to be answered for each video of knee arthroscopy.*

1. How long have you been in practice?
   1. 0 – 5 years
   2. 5 – 10 years
   3. 10 – 20 years
   4. > 20 years
2. How many (not a percentage) meniscus surgeries do you perform per year?
   1. 0 – 10
   2. 10 – 20
   3. 20 – 30
   4. 30 – 40
   5. 40 – 50
   6. > 50
3. How many (not a percentage) DLM do you encounter per year?
   1. 0 – 20
   2. 20 – 40
   3. 40 – 60
   4. 60 – 80
   5. 80 – 100
   6. > 100
4. Would any of these factors influence your DLM post-op restrictions (you can choose more than 1 option)?
   1. Patient’s age
   2. Patient’s BMI
   3. Duration of symptoms
   4. Age of onset
   5. Severity of cartilage lesion
   6. none
5. How much peripheral rim would you try to preserve?
   1. < 3 mm
   2. 3 – 4 mm
   3. 5 – 6 mm
   4. 7 – 8 mm
   5. ≥ 9 mm
   6. I would use the medial meniscus as a reference
   7. Other (please write what you would do)
6. Following saucerization, what would you do?
   1. Nothing else needs to be done
   2. Address meniscus instability
   3. Address meniscus tear
   4. Address meniscus tear + instability
   5. Subtotal meniscectomy
   6. Total meniscectomy
   7. Other (please write what you would do)
7. If you selected repair, your preferred technique would be (if hybrid technique, select more than 1 option):
   1. Inside-out technique
   2. Outside-in technique
   3. All inside technique with capsular implant
   4. All inside technique with knot tying (without implants)
   5. Other (please write what you would do)
   6. No repair needed
8. If you selected repair, how many sutures do you plan on doing?
   1. 1 – 2
   2. 3 – 4
   3. 5 – 6
   4. 7 – 8
   5. 9 – 10
   6. 11 +
   7. No suture needed
9. If you selected meniscus anterior or posterior horn stabilization, your preferred technique would be (if hybrid technique, select more than 1 option):
   1. Anterior capsular stabilization using outside-in technique
   2. Anterior capsular stabilization using all inside technique
   3. Posterior capsular stabilization using inside-out technique
   4. Posterior capsular stabilization using all inside technique
   5. Other (please write what you would do)
   6. No stabilization needed
10. If you selected stabilization, how many sutures do you plan on doing?
    1. 1 – 2
    2. 3 – 4
    3. 5 – 6
    4. 7 – 8
    5. 9 – 10
    6. 11 +
    7. No suture needed
11. Would you use any of the following tissue repair stimulating technique (you can choose more than 1 option)?
    1. Marrow venting with a drill or awl
    2. Injection of autologous conditioned plasma / PRP
    3. Injection of bone marrow aspirated concentrate
    4. Fenestrate the meniscal-capsular junction with a rasp
    5. Trephination using a spinal needle
    6. Usage of fibrin clot or cerclage with collagen membrane
    7. I would not perform any of the above
    8. Other (please write what you would do)
12. Regarding post-op rehabilitation, what are your restrictions regarding weight bearing (please write from week X to week Y)?
    1. Initial non weight bearing:
    2. Initial partial weight bearing:
    3. Initial weight bearing:
13. Regarding post-op rehabilitation, what are your restrictions regarding ROM (please write the different ROM restrictions from week X to week Y)?
14. No initial range of motion
15. Partial range of motion
16. Full range of motion

Appendix 2: Intersurgeon consensus for the different categories of preoperative planning and postoperative restrictions.

| Categories | Questions | VIDEO 1 | VIDEO 2 | VIDEO 3 | VIDEO 4 |
| --- | --- | --- | --- | --- | --- |
|  |  |  |  |  |  |
| Following saucerization | Nothing else needs to be done | 87.5% | 25.0% | 6.3% | 6.3% |
|  | Address meniscus instability | 6.3% | 31.3% | 18.8% | 18.8% |
|  | Address meniscus tear | 0.0% | 0.0% | 25.0% | 25.0% |
|  | address meniscus tear + instability | 6.3% | 43.8% | 50.0% | 50.0% |
|  | Subtotal meniscectomy | 0.0% | 0.0% | 0.0% | 0.0% |
|  | Total meniscectomy | 0.0% | 0.0% | 0.0% | 0.0% |
|  |  |  |  |  |  |
| Peripheral rim preservation | < 3 mm | 0.0% | 0.0% | 0.0% | 0.0% |
|  | 3 – 4 mm | 0.0% | 0.0% | 0.0% | 0.0% |
|  | 5 – 6 mm | 0.0% | 6.3% | 12.5% | 12.5% |
|  | 7 – 8 mm | 31.3% | 31.3% | 31.3% | 31.3% |
|  | 9 + mm | 25.0% | 25.0% | 25.0% | 25.0% |
|  | Use medial meniscus as reference | 12.5% | 12.5% | 12.5% | 12.5% |
|  | Other | 6.3% | 6.3% | 6.3% | 6.3% |
|  | Combined | 25.0% | 18.8% | 12.5% | 12.5% |
|  |  |  |  |  |  |
| Meniscus repair technique | Inside-out | 0.0% | 0.0% | 12.5% | 18.8% |
|  | Outside-in | 0.0% | 12.5% | 0.0% | 0.0% |
|  | All inside + capsular implant | 6.3% | 0.0% | 25.0% | 18.8% |
|  | All inside + knot tying | 0.0% | 6.3% | 6.3% | 6.3% |
|  | Other | 0.0% | 0.0% | 0.0% | 6.3% |
|  | Combined | 6.3% | 31.3% | 31.3% | 18.8% |
|  | No repair | 87.5% | 50.0% | 25.0% | 31.3% |
|  |  |  |  |  |  |
| Suture for meniscus repair | 1 – 2 | 0.0% | 6.3% | 6.3% | 0.0% |
|  | 3 – 4 | 6.3% | 25.0% | 31.3% | 25.0% |
|  | 5 – 6 | 0.0% | 12.5% | 18.8% | 31.3% |
|  | 7 – 8 | 6.3% | 6.3% | 18.8% | 6.3% |
|  | 9 – 10 | 0.0% | 0.0% | 0.0% | 0.0% |
|  | 11 + | 0.0% | 0.0% | 0.0% | 6.3% |
|  | No suture | 87.5% | 50.0% | 25.0% | 31.3% |
|  |  |  |  |  |  |
| Meniscus instability technique | Anterior Outside-in | 0.0% | 37.5% | 0.0% | 0.0% |
|  | Anterior all inside | 0.0% | 0.0% | 0.0% | 0.0% |
|  | Posterior Outside-in | 0.0% | 0.0% | 6.3% | 6.3% |
|  | Posterior All-inside | 6.3% | 6.3% | 43.8% | 12.5% |
|  | Other | 0.0% | 12.5% | 6.3% | 6.3% |
|  | Combined | 6.3% | 18.8% | 12.5% | 43.8% |
|  | No instability | 87.5% | 25.0% | 31.3% | 31.3% |
|  |  |  |  |  |  |
| Suture for meniscus instability | 1 – 2 | 0.0% | 25.0% | 25.0% | 6.3% |
|  | 3 – 4 | 6.3% | 25.0% | 25.0% | 25.0% |
|  | 5 – 6 | 0.0% | 18.8% | 6.3% | 25.0% |
|  | 7 – 8 | 6.3% | 6.3% | 12.5% | 6.3% |
|  | 9 – 10 | 0.0% | 0.0% | 0.0% | 0.0% |
|  | 11 + | 0.0% | 0.0% | 0.0% | 0.0% |
|  | No suture | 87.5% | 25.0% | 31.3% | 37.5% |
|  |  |  |  |  |  |
| Repair stimulating technique | Marrow venting | 0.0% | 18.8% | 25.0% | 31.3% |
|  | Plasma rich platelets | 0.0% | 6.3% | 6.3% | 6.3% |
|  | Injection of bone marrow | 0.0% | 0.0% | 0.0% | 0.0% |
|  | Fenestrate + rasp | 0.0% | 6.3% | 6.3% | 0.0% |
|  | Trephination | 0.0% | 6.3% | 6.3% | 0.0% |
|  | Fibrin clot or cerclage | 0.0% | 0.0% | 0.0% | 0.0% |
|  | None | 93.8% | 18.8% | 12.5% | 12.5% |
|  | Combined | 6.3% | 43.8% | 43.8% | 50.0% |
|  |  |  |  |  |  |
| Weightbearing | Initial non weightbearing | 6.3% | 25.0% | 31.3% | 25.0% |
|  | Initial partial weightbearing | 0.0% | 37.5% | 31.3% | 37.5% |
|  | Initial full weightbearing | 93.8% | 37.5% | 37.5% | 37.5% |
|  |  |  |  |  |  |
| Range of motion | No range of motion | 0.0% | 6.3% | 6.3% | 6.3% |
|  | Partial range of motion | 6.3% | 43.8% | 62.5% | 62.5% |
|  | Full range of motion | 93.8% | 50.0% | 31.3% | 31.3% |

Video 1 corresponds to (W2;H0;S0;T0), video 2 corresponds to (W2;H0;SA;T0), video 3 corresponds to (W2;H1;S0;THAP) and video 4 corresponds to (W2;H1;SP;THAP).
